# Acute angle closure glaucoma secondary to polypoidal choroidal vasculopathy – a devastating complication

**DOI:** 10.3205/oc000052

**Published:** 2017-01-05

**Authors:** Prabu Baskaran, Manavi D. Sindal, Pankaja Dhoble, Seema Ramakrishnan, Venkatesh Rengaraj, Pradeep Ramulu

**Affiliations:** 1 Aravind Eye Hospital and Postgraduate Institute of Ophthalmology, Pondicherry, India; 2 Wilmer Eye Institute, John Hopkins University, Baltimore, USA

**Keywords:** angle closure glaucoma (ACG), hemorrhagic choroidal detachment (CD), polypoidal choroidal vasculopathy (PCV)

## Abstract

Acute angle closure glaucoma (ACG) in the setting of polypoidal choroidal vasculopathy (PCV) is a catastrophic complication that has been documented infrequently in literature. Ours is the second only report that describes hemorrhagic choroidal detachment as an event leading to acute angle closure glaucoma in PCV patients and the first one to describe the use of diode cyclophotocoagulation (CPC) for this condition. The purpose of this article is to familiarize readers with this entity that has an extremely dismal visual prognosis. Ours is a descriptive case report of two patients with PCV complicated by sudden onset hemorrhagic choroidal detachment (CD) and acute ACG. Both patients had severe pain with no perception of light at presentation with an acute angle closure attack. Both underwent diode CPC for pain relief and control of intraocular pressure (IOP). Both our patients did not regain any vision, but their pain was relieved by diode CPC. Both eyes eventually became phthisical. Acute ACG following massive hemorrhagic CD is a rare but grave complication of PCV, not amenable to treatment. Diode CPC is an effective palliative modality of management to achieve pain relief in such cases.

## Introduction

The initial description of polypoidal choroidal vasculopathy (PCV) was given by Yanuzzi [1] in 1982 as polypoidal, subretinal, vascular lesions associated with serous and hemorrhagic detachments of retinal pigment epithelium (RPE). Later in the year 1984, Kleiner et al. [2] observed a similar condition in a group of middle-aged African-American women who had a peculiar hemorrhagic disorder of the macula characterized by recurrent subretinal and sub-RPE bleeding. He termed this as posterior uveal bleeding syndrome. Polypoidal choroidal vasculopathy can occur in both sexes and is generally seen in the elderly [3]. PCV is a disease of the posterior segment, and though indocyanine green angiography (ICGA) is the gold standard for the diagnosis of PCV, optical coherence tomography (OCT) is emerging as a very useful tool in identifying this condition. De Salvo et al. have shown a sensitivity of 94.6% and a specificity of 92.9% in identifying PCV by OCT [4]. There had been no standard treatment guidelines for PCV before the EVEREST trial was published in September 2012. This landmark trial has shown that both verteporfin monotherapy and verteporfin and ranibizumab combination therapy are superior to ranibizumab monotherapy in achieving complete polyp regression [5]. Acute angle closure glaucoma as sequelae of PCV is very rare and has been reported only once before in literature [6]. We report herein two cases of this rare complication that we managed with palliative treatment.

## Case descriptions

### Case 1

**Figure 1:**
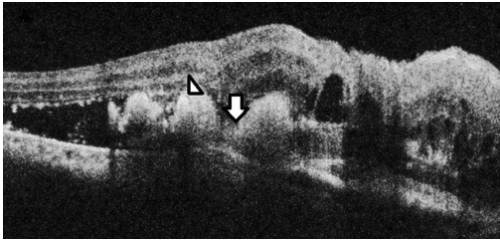
OCT of the right eye showing multiple hemorrhagic PEDs with notch (arrow) and underlying polyps (arrow head) surrounded by SRF

**Figure 2:**
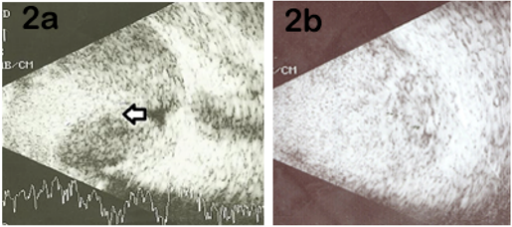
(2a) B scan ultrasonography of case 1 showing annular hemorrhagic CD with “kissing choroids” (arrow); (2b) B scan of case 2 showing findings similar to 2a

A 67-year-old female presented in July 2012 with defective vision in the right eye for six months. She was a known hypertensive and diabetic on treatment for both conditions for the last 15 years. There was no history of trauma, blood dyscrasias or use of antiplatelet or anticoagulant therapy. On examination, her best-corrected visual acuity (BCVA) was counting fingers at face in the right eye and 6/6 in the left eye. Intraocular pressure (IOP) by non contact tonometry (NCT) was within normal limits in both eyes. Anterior segment examination was unremarkable except for bilateral pseudophakia. Fundus examination of the right eye showed three hemorrhagic pigment epithelial detachments (PED) surrounded by serosanguinous fluid, while the fundus in the left eye was within normal limits. OCT of the right eye showed notched hemorrhagic PEDs with underlying polyps and surrounding subretinal fluid (SRF) suggestive of PCV (Figure 1 (Fig. 1)). Based on the clinical picture and OCT findings, a diagnosis of PCV was made and the patient was treated with three loading doses of intravitreal bevacizumab at monthly intervals. After three injections, OCT showed evidence of scarring and resolution of SRF. Best corrected visual acuity improved to 2/60. The patient was kept under observation at monthly intervals for any signs of activity such as drop in vision, SRF with or without intraretinal fluid, PED and subretinal hemorrhage [7]. She followed up with us on a monthly basis till February 2013, thereafter she was lost to follow-up. After a gap of four months, she presented again to us in June 2013 with complaints of swelling, pain, and redness of the right eye since nine days. At this visit, vision in the right eye was no perception of light (NPL) and IOP was 50 mm Hg by applanation tonometry. Anterior segment examination showed lid edema, conjunctival chemosis, corneal epithelial edema, blood staining of cornea, a 3 mm hyphema, and a shallow anterior chamber. Corneal edema precluded a view of fundus and gonioscopy revealed closed angles in the superior, nasal and temporal angles that were unobscured by hyphema. B Scan ultrasonography showed annular appositional hemorrhagic choroidal detachment (Figure 2a (Fig. 2)). The left eye was within normal limits. In order to reduce the pain and IOP, palliative 360° diode cyclophotocoagulation (CPC) was performed. Within four months of diode CPC, the eye became phthisical and the patient has been on regular follow-up till date without any discomfort.

### Case 2

A 71-year-old female known diabetic and hypertensive over the last 20 years presented in February 2012 with the complaints of poor vision in the left eye for the past three months. She was diagnosed to have PCV on ICGA done at an outside institution. On examination, her BCVA was 6/12 in the right eye and 6/36 in the left eye. IOP was within normal limits. Anterior segment examination in the right eye showed anterior chamber intraocular lens and the left eye showed nuclear sclerosis grade III. Fundus examination revealed moderate non proliferative diabetic retinopathy in the right eye and a large parafoveal subretinal hemorrhage with hemorrhagic PED in the left eye suggestive of PCV. The patient underwent phacoemulsification with posterior chamber intraocular lens (PCIOL) implantation in March 2012 in the left eye. Following this, ICGA-guided PDT with ranibizumab was planned, but before she could undergo this treatment, she developed an acute episode of pain with vomiting in April 2012. Her visual acuity at this visit was NPL and applanation tension was 44 mm Hg. Slit lamp examination showed epithelial corneal edema, shallow anterior chamber, PCIOL, and hemorrhagic CD in the anterior vitreous face. Anterior chamber angles were closed on gonioscopy and B Scan ultrasonography revealed annular hemorrhagic CD with ‘kissing choroids’ (Figure 2b (Fig. 2)). The patient was initially managed with anti-glaucoma medications, topical steroids and cycloplegics. Since the pain was not controlled with conservative treatment, she underwent diode CPC. Though there was relief from pain following diode CPC, the eye became phthisical after two months. She has since been on regular follow-up without any discomfort. 

## Discussion

Spontaneous massive subretinal hemorrhage is a known complication of PCV arising from rupture of thin walled choroidal vessels. Polypoidal choroidal vasculopathy causing massive suprachoroidal bleed and appositional hemorrhagic CD resulting in secondary angle closure glaucoma has been reported only once before [6]. Table 1 (Tab. 1) shows a concise review of literature on nine cases including our two that had acute secondary angle closure glaucoma due to a large spontaneous subretinal or suprachoroidal bleed [6], [8], [9], [10] . Only one of the other seven cases is secondary to PCV [6]. Four cases belonging to a series reported by Chen et al. had angle closure glaucoma secondary to age-related macular degeneration [10]. Though various treatment modalities such as anti-glaucoma medications, laser peripheral iridotomy and iridoplasty, sclerotomy, etc. have been tried to control the refractory glaucoma, the final outcome in almost all cases has been poor. Seven of nine eyes became phthisical while one was enucleated. In both our patients, diode CPC was used as an effective tool for controlling pain. 

The proposed mechanism for secondary angle closure is that annular hemorrhagic CD can result in the forward displacement of the lens-iris diaphragm causing an angle closure attack [8], [9]. The predisposing factors for a suprachoroidal bleed include oral anticoagulant therapy, blood dyscrasias, and systemic hypertension [9]. Our patients did not have any predisposing risk factors other than systemic hypertension, which in both cases was well controlled. The visual prognosis of patients with angle closure glaucoma due to appositional hemorrhagic CD is very poor despite timely medical and surgical management. We have reported two cases of this rare complication of PCV. We wish to emphasize the fact that appositional hemorrhagic CD with secondary angle closure glaucoma in the setting of PCV can lead to irreversible blindness. Diode CPC is an effective palliative modality of management to achieve pain relief in such cases.

## Notes

### Competing interests

The authors declare that they have no competing interests.

## Figures and Tables

**Table 1:**
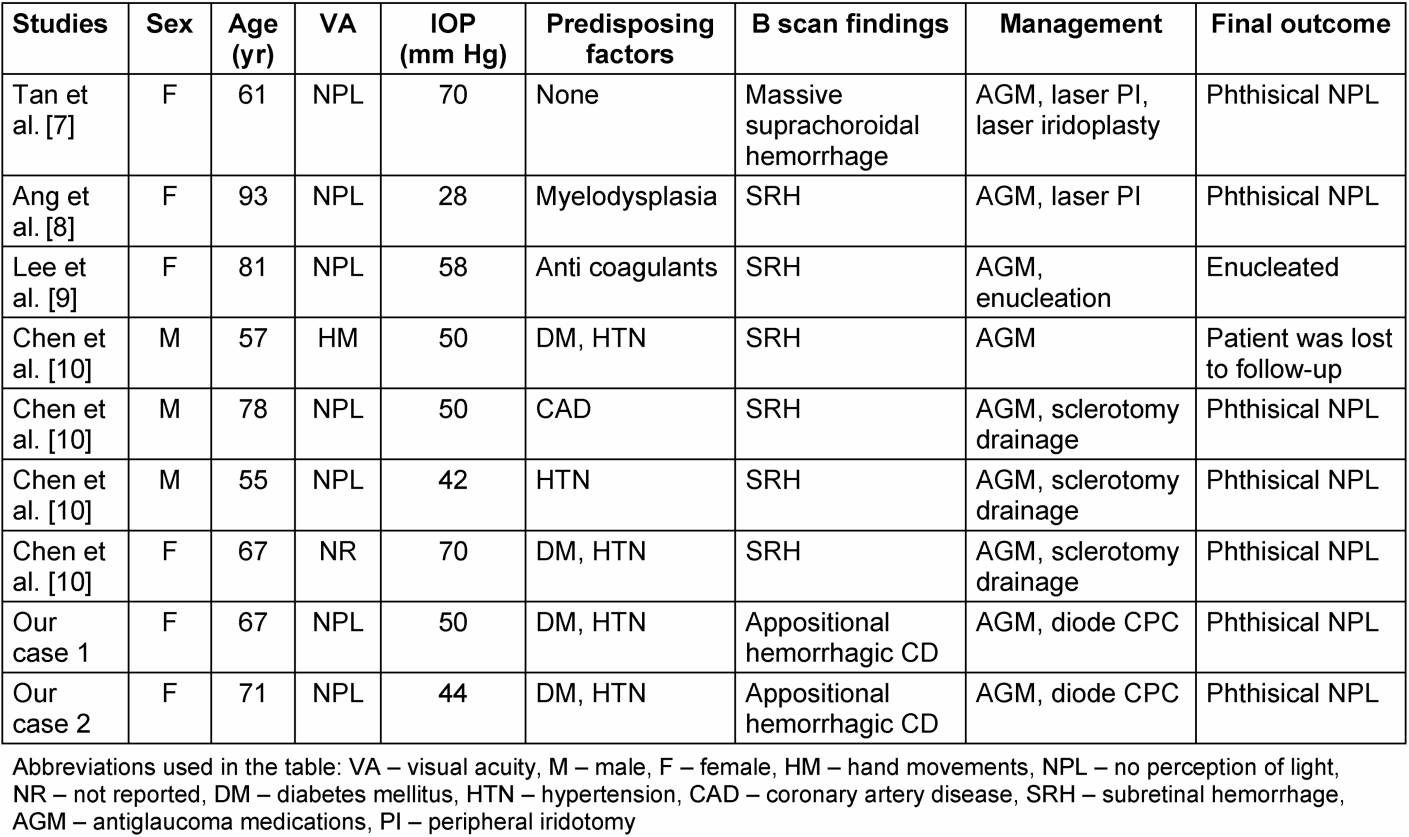
Review of literature on PCV/AMD/other related cases complicated by secondary ACG
